# Evaluating a collaborative smoking cessation intervention in primary care (ENTER): study protocol for a cluster-randomized controlled trial

**DOI:** 10.1186/s13063-015-0977-6

**Published:** 2015-10-09

**Authors:** Martin Härter, Anna-Lena Bartsch, Nina Egger, Hans-Helmut König, Levente Kriston, Holger Schulz, Michael Tiemann, Anna Levke Brütt, Angela Buchholz

**Affiliations:** Department of Medical Psychology, University Medical Center Hamburg-Eppendorf, Martinistraße 52, 20246 Hamburg, Germany; Department of Health Economics and Health Services Research, University Medical Center Hamburg-Eppendorf, Martinistraße 52, 20246 Hamburg, Germany; Department of Prevention and Health Promotion, AOK NORDWEST, Kopenhagener Straße 1, 44269 Dortmund, Germany

**Keywords:** Cardiovascular disease, Chronic obstructive pulmonary disease, Health services research, Primary medical care, Smoking cessation

## Abstract

**Background:**

Tobacco consumption is a preventable risk factor for chronic disease and complicates the treatment of medical conditions. Therefore, the German health insurance company AOK NORDWEST has developed a collaborative smoking cessation intervention for individuals with cardiovascular disease, chronic obstructive pulmonary disease and heavy smokers, with the aim of reducing tobacco consumption. The objective of the study ENTER is to evaluate the effectiveness of the collaborative smoking cessation intervention and determine its cost-effectiveness.

**Methods/Design:**

This study is a cluster-randomized controlled trial conducted with 40 medical practices that are being selected from different geographic regions in Germany. Participating medical practices will be randomly allocated to either the intervention or control group. Within the medical practices, a total of 800 patients will be recruited for participation in the study and blinded to group assignment. Patients are included in the study if they are 18 years or older, insured by AOK, heavy smokers (smoke at least 20 cigarettes per day) and/or suffer from chronic obstructive pulmonary disease or cardiovascular disease. Exclusion criteria are patients who are nonsmokers, who have cognitive impairments or who are illiterate. Physicians from medical practices in the intervention group will motivate patients to participate in a smoking cessation program offered by the health insurance, refer them to the program and ask about their program participation. Physicians from medical practices in the control group will provide usual care. Data collection will take place on the date of study inclusion and after 6 and 12 months. The primary outcome is the amount of cigarettes consumed during the past 30 days, 12 months after the initial medical consultation. Secondary outcomes are abstinence from smoking, health-related quality of life and respiratory complaints. Moreover, a process evaluation and health economic analysis will be performed.

**Discussion:**

The results of this study will help to determine whether the collaborative smoking cessation intervention is an effective and feasible way to promote smoking cessation in the primary care setting and provide evidence regarding its cost-effectiveness.

**Trial registration:**

German Clinical Trials Register DRKS00006079. Registered 4 June 2014.

## Background

In Germany, around 29 % of the adult population currently smoke, and 25 % of these smokers consume 20 cigarettes or more per day [1]. The negative health effects associated with regular tobacco consumption are well known: tobacco consumption is a risk factor for cancer, cardiovascular and respiratory diseases [2]. Furthermore, tobacco consumption complicates the treatment of many chronic diseases. Therefore, key elements in the treatment and secondary prevention of chronic obstructive pulmonary disease (COPD) [3] and coronary diseases are to avoid exposure to tobacco smoke [4]. Within the first year, abstinence from tobacco leads to an improvement in the lung function of COPD patients as measured by Forced Expiratory Volume in 1 second (FEV1). The progressive decrease in FEV1 can be stopped in the further course of treatment [5]. As a form of secondary prevention, smoking cessation reduces the risk of mortality associated with smoking-related diseases [6]. Moreover, a 50 % reduction in cardiovascular events is expected as a result of tobacco cessation [7]. Consequently, a number of different strategies to prevent tobacco consumption have been incorporated into prevention guidelines [8].

There are a number of different smoking cessation methods such as self-help measures, pharmacological aids, alternative treatments and individual or group counseling [9]. In contrast to pharmacological aids, certified smoking cessation programs are reimbursed in Germany. The smoking cessation program “Rauchfrei Programm” developed by the Institute for Therapy Research (IFT) in Munich and the German Federal Center for Health Education (Bundeszentrale für gesundheitliche Aufklärung) is most frequently offered by German health insurance companies [10]. The program consists of 3 to 7 weekly meetings with up to 12 participants and individual, telephone-based counseling [3]. The program is based on a cognitive-behavioral approach with the aim of creating problem awareness, motivating and achieving attitude as well as behavior change. Data from an evaluation (single group, pre-post-follow-up design) of the smoking cessation program in a routine setting with 1,319 participants demonstrate that the program is effective [11]. On average, participants smoked 20 cigarettes per day at the beginning of the program (*SD = 9)* and were heavily nicotine-dependent [11]. The abstinence rate was 61 %, and 32 % after 1 year [11].

Despite the availability of smoking cessation programs, few smokers participate in the programs that are currently offered. Only 1 % of the prevention programs covered by public health insurances (e.g. physical exercise, stress management and nutrition) are in the field of substance abuse which mainly consists of smoking cessation programs (96 %) [12]. The low uptake of smoking cessation programs indicates that there is a need for innovative and networked strategies that motivate smokers and individuals who suffer from smoking-related diseases, such as cardiovascular disease and COPD, to quit smoking [13]. For example, besides extending and improving smoking cessation programs, there is a need to disseminate more information about available programs and to facilitate access to them [14]. This form of health promotion is based on the New Public Health approach, which proposes that interventions should be implemented on a behavioral as well as a structural level [15]. According to this approach, health behavior change does not only depend on the individual person but also on the social, economic and political environment.

General practitioners (GPs) play an important part in facilitating access to smoking cessation programs because they are consulted by 70 % of smokers at least once per year [16]. As pulmonologists and internists situated in individual offices are also frequently consulted by smokers, they are considered equally important. For the purpose of clarity, GPs, pulmonologists and internists are referred to only as physicians in the following. Due to the frequent patient contact, the physician’s office can be used to support and motivate smokers to participate in smoking cessation programs. German smoking cessation guidelines recommend that physicians ask every patient about their smoking behavior and provide smoking cessation advice [17]. This approach is warranted because a number of studies have shown that smoking cessation advice offered by physicians increases quit rates [7].

Therefore, the health insurance company AOK NORDWEST (referred to as AOK in the following) has developed a Collaborative Smoking Cessation Intervention (CSCI), which builds on these findings and is directed at individuals with cardiovascular disease or COPD and heavy smokers. The CSCI concept is derived from an earlier project by AOK aimed at reducing risk factors associated with a lack of physical exercise, which has been successfully implemented in Westphalia-Lippe [18]. The aim of CSCI is to decrease the rate of tobacco consumption among individuals with cardiovascular disease, COPD and heavy smokers, in order to improve quality of life and overall therapeutic success.

### Study aim and hypotheses

The aim of the present study is to evaluate the effectiveness and cost-effectiveness of CSCI with respect to tobacco consumption, health status and health care costs, compared to usual care. The main hypotheses are listed.

The primary hypothesis is as follows:

Patients in the intervention group smoke fewer cigarettes per day (30-day prevalence) compared to patients in the control group.

The secondary hypotheses are as follows:

1. Patients in the intervention group are abstinent from smoking more often compared to patients in the control group.
2. Patients in the intervention group have higher health-related quality of life compared to patients in the control group.
3. Patients in the intervention group have fewer respiratory complaints, compared to patients in the control group.

The hypotheses related to the process evaluation are as follows:

1. The amount of patients referred to the smoking cessation program is greater in the intervention group compared to the control group.
2. The amount of patients who attend the smoking cessation program is greater in the intervention group compared to the control group.
3. Cost-effectiveness (cost per reduced quantity-frequency index) and cost-utility (cost per quality-adjusted life year; QALY) are greater in the intervention group compared to the control group.

## Methods/Design

### Study design

A two-arm, cluster-randomized controlled trial will be conducted with approximately 40 medical practices, selected from different regions in Germany (Westphalia-Lippe and Schleswig-Holstein). Patients from these medical practices will be assessed on the date of study inclusion (t0) and after 6 (t1) and 12 months (t2). Cluster-randomization is used with medical practices chosen as the unit of allocation because it is most practical in this setting and minimizes the risk of spillover effects from the intervention to the control group [7]. Moreover, the collaboration between physicians, their medical assistants and AOK will be assessed via qualitative interviews conducted 12 months after study begin. Finally, the intervention’s cost-effectiveness will be assessed.

### Recruitment and randomization

#### Recruitment of physicians

Letters of invitation to participate in the study will be sent to physicians situated in individual medical practices by AOK. Letters will be followed up via telephone by a research assistant from the University Medical Center Hamburg-Eppendorf, in case of no response. Physicians who agree to participate in the study via written consent will be randomly assigned to the intervention or control group (the intended allocation ratio is 1:1).

### Randomization

Physicians participating in the study will be stratified by geographic region and randomly allocated to either the intervention or control group by means of permuted block-randomization with randomly varying block sizes (blocks of two and four). The trial statistician will generate a random number sequence via computer on the basis of which one envelope will be created for each medical practice with the result of the allocation. When a medical practice is included in the study, a research assistant without prior knowledge of the sequence will open the next envelope (one at a time) and allocate the medical practice to the intervention or control group.

### Recruitment of patients

Within participating medical practices, patients will be recruited for participation in the study and blinded to group assignment. In order to participate in the study, patients must be 18 years or older, insured by AOK, heavy smokers (smoke at least 20 cigarettes per day) and/or suffer from COPD or cardiovascular disease. Exclusion criteria are nonsmokers and patients who have cognitive impairments or are illiterate.

### Measures and evaluation

#### Measures

Patients are handed a questionnaire (t0) at the medical practice containing the quantity-frequency index [19], the Fagerström Test for Nicotine Dependence (FTND) [20], SmoCess-GP [21], Questionnaire to assess readiness to change among smokers (FÄR) [22], the Short Form SF-12 [23], the EQ-5D-3L [24] and the Visual Simplified Respiratory Questionnaire (VSRQ) [25]. Physicians specify the patient’s smoking status and the diagnosis for the severity of the cardiovascular disease or COPD on a documentation sheet. Follow-up takes place after 6 (t1) and 12 (t2) months via telephone by a research assistant from the University Medical Center Hamburg-Eppendorf. Moreover, qualitative interviews will be conducted with physicians assigned to the intervention group, their medical assistants and the AOK smoking cessation program instructors after 12 months in order to determine the network’s quality. The measures used in this study are validated and have been used previously in similar studies. All measures are listed in Table 1 along with the time of measurement and person being assessed.

**Table 1:** Measurement process and time

Time of measurement	Person	Constructs
Screening	Patient	Smoking status (>20 cigarettes per day yes/no) and/or presence of cardiovascular disease or COPD
Questionnaire (t0)	Patient	Smoking status:
		Fagerström Test for Nicotine Dependence (FTND) [20]
		Quantity-frequency index (30 days): Amount of days on which patients smoked during the last 30 days, average amount of cigarettes consumed per day [19]
		SmoCess-Questionnaire to assess primary care for smoking cessation [21]
		Health status:
		Health-related quality of life SF-12 [23]
		EQ-5D-3L [24]
		Respiratory complaints: Visual Simplified Respiratory Questionnaire (VSRQ) [25]
		Motivation:
		Questionnaire to assess readiness to change among smokers (FÄR) [22]
Documentation sheet (t0)	Physician	Smoking history (number of years of smoking), relevant clinical indications (severity of cardiovascular disease or COPD)
Questionnaire (t1)	Patient	Smoking status:
		Fagerström Test for Nicotine Dependence (FTND) [20]
		Quantity-frequency index (30 days): Amount of days on which patients smoked during the last 30 days, average amount of cigarettes consumed per day [19]
		SmoCess-Questionnaire to assess primary care for smoking cessation [21]
		Health status:
		Health-related quality of life SF-12 [23]
		EQ-5D-3L [24]
		Respiratory complaints: Visual Simplified Respiratory Questionnaire (VSRQ) [25]
		Motivation:
		Questionnaire to assess readiness to change among smokers (FÄR) [22]
Questionnaire (t2)	Patient	Smoking status:
		Fagerström Test for Nicotine Dependence (FTND) [20]
		Quantity-frequency index (30 days): Amount of days on which patients smoked during the last 30 days, average amount of cigarettes consumed per day [19]
		SmoCess-Questionnaire to assess primary care for smoking cessation [21]
		Health status:
		Health-related quality of life SF-12 [23]
		EQ-5D-3L [24]
		Respiratory complaints: Visual Simplified Respiratory Questionnaire (VSRQ) [25]
		Motivation:
		Questionnaire to assess readiness to change among smokers (FÄR) [22]

### Evaluation

#### Outcome evaluation

Primary and secondary outcomes are assessed on the individual participant level 12 months after the initial consultation with the physician. The primary outcome is the amount of cigarettes smoked per day (30-day prevalence), measured via the quantity-frequency index [19]. Secondary outcomes are the rate of abstinence from smoking, quality of life measured via sum scores calculated from the SF-12 [23], and respiratory complaints measured via sum scores calculated from the Visual Simplified Respiratory Questionnaire [25].

### Process evaluation

The process evaluation is performed at the cluster-level and based on semi-structured telephone interviews that will be conducted with physicians assigned to the intervention group, their medical assistants and AOK course instructors 12 months after inclusion into the study. During the interviews, a focus will be placed on perceived barriers and facilitators to the implementation of the intervention. Further questions will concern experiences with implementing the intervention under routine conditions, the collaboration with AOK, organizational matters and perceived advantages of the intervention. In addition, physicians will be asked about their experiences with motivating patients and AOK course instructors will be asked about the participation of patients during the courses. The amount of patients participating in smoking cessation courses will also be assessed. Moreover, the health economic analysis will serve to determine the intervention’s cost-effectiveness (cost per reduced quantity-frequency index) and cost-utility (cost per quality-adjusted life year; QALY) from the perspective of the German statutory health insurance. The analyses will be performed with AOK routine data covering the period 12 months before until 12 months after the intervention. Routine data will contain information about the use of health care services and related costs due to hospital care, outpatient medical and psychotherapeutic care by physicians and specialists, medication, medical aids, medical rehabilitation, inability to work and sickness allowance. The costs of the intervention will be calculated per patient following a micro-costing. This will include the compensation physicians receive per recommendation as well as costs resulting from the provision of the intervention, i.e. remuneration of course instructors, course materials and room rent.

### Study procedure

Medical assistants will screen patients for inclusion in the study by means of a checklist. Patients who meet the inclusion criteria will be informed about the study via a study information. Patients who wish to participate in the study will be asked to provide their contact details on a separate form and complete the first questionnaire (t0) during the waiting time, along with the informed consent form and a contact information sheet. Patients will be asked to return the questionnaire to a medical assistant in a sealed envelope together with the informed consent form and contact information sheet before leaving the medical practice. Informed consent will be obtained from all patients participating in the study. Medical assistants will keep a list of all patients who were informed about the study and a separate list of patients who participate in the study. Physicians will specify patient’s smoking status and the severity of cardiovascular disease/COPD on a documentation sheet. At the end of the survey phase, the lists and documentation sheets will be sent to the study center at the University Medical Center Hamburg-Eppendorf. A 6-month (t1) and 12-month (t2) follow-up will be carried out via telephone by a research assistant using the information provided by patients on the contact information sheet. Moreover, the collaboration between physicians, their medical assistants and AOK staff will be assessed via qualitative interviews conducted 12 months after the start of the study by a research assistant. Telephone interviews will be recorded digitally, transcribed and analyzed via qualitative content analysis [26]. An evaluation of the intervention’s cost effectiveness will also be performed.

### Collaborative smoking cessation intervention (CSCI)

Physicians in the CSCI group will receive a brochure containing detailed information about the intervention, the steps involved in delivering it to patients and the required materials. The steps involved in delivering the intervention are as follows: During a personal consultation physicians motivate patients to participate in the smoking cessation program. Patients who wish to participate in the program are handed a flyer containing detailed information about the date, time and place of the program and are instructed to contact the nearest AOK branch office to register for the program. Patients who fail to do so within 14 days are sent a written reminder by AOK. Physicians issue a referral form for each patient willing to participate in the program and send it to AOK. Physicians receive a one-time payment of €25.50 from AOK for every patient referred to the smoking cessation program via referral form. During subsequent consultations, physicians ask patients about their participation in the program. Physicians assigned to the control group will provide usual care (defined as the care provided by physicians under routine conditions, when not trained to deliver the CSCI).

### Statistical methods

#### Data analysis

The main research questions will be evaluated via mixed models, which make it possible to examine effects and their interactions on different levels (physicians and patients). According to the intent-to-treat principle, all randomized patients will be included in the primary analyses. In the analysis of abstinence rates, patients lost to follow-up will be assumed to have returned to smoking [27, 28]. Table 2 provides a summary of the hypotheses along with comparison groups, statistical method and the outcomes of interest. Other than the 6-month follow-up conducted with patients via telephone, no interim analyses are planned.

**Table 2:** Hypotheses

Hypotheses	Comparison	Statistical method	Outcome measure
Primary hypothesis			
Smoke reduction	CG-IG	Mixed models	Quantity-frequency index
Secondary hypotheses			
Rate of abstinence	CG-IG	Chi-square	Abstinence
Health-related quality of life	CG-IG	Mixed models	SF-12
Respiratory complaints	CG-IG	Mixed models	Visual Simplified Respiratory Questionnaire
Process evaluation hypotheses			
Referral	CG-IG	Descriptive	Amount of patients referred to the smoking cessation program via referral form
Uptake	CG-IG	Descriptive	Amount of patients attending the smoking cessation program
Cost-effectiveness and cost-utility	CG-IG	ICER	Quantity-frequency index, QALY

The telephone interviews conducted with physicians and AOK staff will be recorded digitally, transcribed and analyzed via qualitative content analysis [26]. The health economic analysis will be performed by calculating the incremental cost-effectiveness ratio (ICER) for the 12-month follow-up, based on routine cost data provided by AOK. As measures of effects, the quantity frequency index, as well as QALYs, will be employed. For this purpose, differences in the mean costs ($\overline{\boldsymbol{C}}$) and mean effects (***Ē***) between intervention and control group will be related to one another:

$$ ICER=\frac{{\overline{C}}_{IG}-{\overline{C}}_{CG}}{{\overline{E}}_{IG}-{\overline{E}}_{CG}}=\frac{\varDelta \overline{C}}{\varDelta \overline{E}} $$

The nonparametric bootstrap procedure will be used to perform the ICER’s uncertainty analysis. This procedure takes into account the skewness of cost data and the covariance of costs and QALYs. In order to control for possible confounding variables and to account for clustering, an alternative procedure (Net-Benefit Regression) will be used. Cost-effectiveness acceptability curves will be created in order to illustrate the statistical uncertainty.

### Sample size calculation

**Fig. 1:**
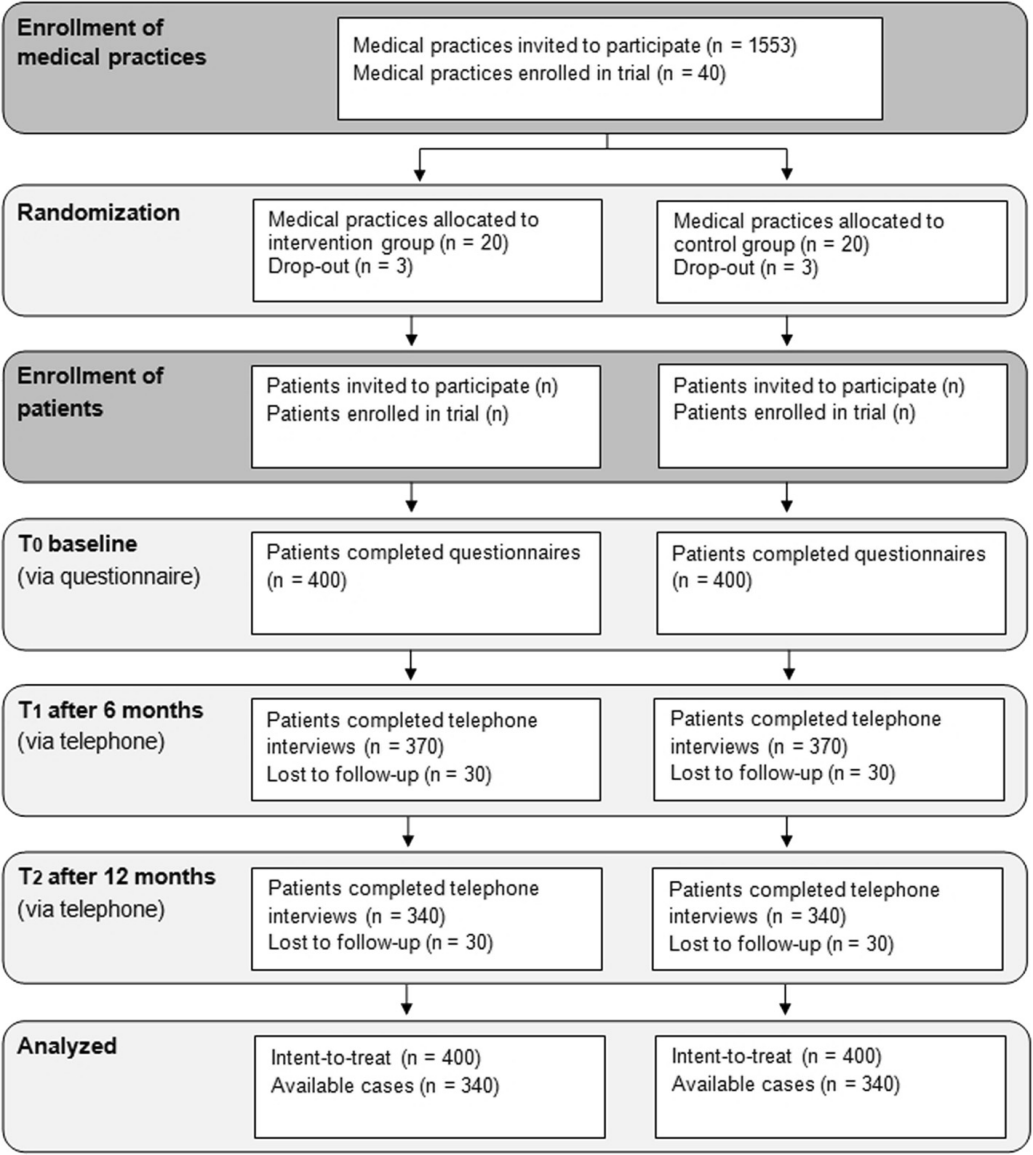
Flow chart illustrating the expected flow of medical practices and patients through the study

According to a survey of 11,000 physicians, on average, 53 patients are treated in medical practices per day [29]. Approximately 15 of these patients are health insured by AOK, which holds one third of the nationwide market share. According to epidemiological data, approximately 7 % of patients smoke 20 or more cigarettes per day. Assuming a participation rate of 50 %, physicians should be able to recruit around 10 patients for participation in the study per month. A meta-analysis by Stead and colleagues [7] demonstrates that among at-risk patients, the relative risk of being abstinent from smoking at follow-up is 1.65 for participants of intensive interventions, compared to participants of minimal interventions (with around 12 and 7 % cessation rates in the intervention and control groups, respectively). This value was transformed into a standardized mean difference (Cohen’s d) due to the metric scaling of our primary outcome. The expected small to moderate effect of Cohen’s d = 0.30 requires that data from 352 persons are available for sufficiently powered (β = 0.20) independent samples t-tests (α = 0.05). Accounting for the effect of clustering (intraclass correlation coefficient of 0.05) [30, 31]suggests that data from 34 medical practices with an average cluster size of 20 should be analyzed (340 patients per group, 680 in total). An estimated 15 % drop-out rate for medical practices means that 40 medical practices should be recruited. Due to an estimated 15 % of patients lost to follow-up, a total of 800 patients will be recruited for participation in the study. Assuming a 20 % participation rate in the smoking cessation program, 68 patients from the intervention group are expected to participate in the smoking cessation program. Figure 1 illustrates the expected flow of medical practices and patients through the study.

### Ethical considerations

This research will be conducted in accordance with the Declaration of Helsinki and ICH Good Clinical Practice guidelines. The study has been designed in consultation with the AOK data protection officer and has been approved by the ethics committees of the medical associations in Hamburg (Reg.-No. PV4628), Westphalia-Lippe (Reg.-No. 2014-326-b-S) and Schleswig-Holstein (Reg.-No. IV/EK189/14). The program “Programm Rauchfrei” is well established and side effects are not known, so that adverse events or ethical concerns are not expected.

A participant information statement serves to inform all patients about the study’s aim, procedure and data management. Moreover, patients will be given an informed consent form and asked to complete it. Patients will also be asked to provide their contact details (name and telephone number) on a contact information sheet, so that a research assistant from the University Medical Center Hamburg-Eppendorf can contact them for the purpose of conducting follow-up interviews. Until follow-up interviews have been completed, pseudonyms will be used and the participant’s contact details will be stored separately from questionnaire or interview data and the documentation sheet. Access to the final dataset will only be granted to authorized staff at the University Medical Center Hamburg-Eppendorf. Collaborating institutions will be required to agree to data management procedures so that access is restricted contractually. Participation in the study is voluntary and can be withdrawn at any time by patients without facing consequences or having to give an explanation. In case patients withdraw from the study, their data will be deleted.

## Discussion

### Purpose and use of the results

Funding innovative approaches in the field of prevention depends on their demonstrated effectiveness according to current guidelines [17]. The aim of this study is to evaluate the effectiveness of CSCI [32]. CSCI builds on previous research, which has shown that smoking cessation advice offered by physicians increases quit rates [33] and that appropriate training leads physicians to ask patients about their smoking status [34]. The focus of CSCI is laid on establishing local networks between AOK and physicians. Therefore, the study results will help to determine whether this is a suitable approach to support smoking cessation in the primary care setting.

Moreover, the results will provide information regarding the feasibility of implementing CSCI under routine conditions. This information is valuable because CSCI is based on current guidelines, which recommend that physicians ask every patient about their smoking status [17]. The results of this study are of further policy relevance because they can inform the design of future behavioral and structural level interventions in the field of health services. Furthermore, the findings of the economic evaluation will provide insight concerning whether the intervention proves cost-effective and whether medical cost savings can be achieved in the short-term.

Generalizability of the study results may be limited because physicians and patients willing to participate in a study on smoking cessation could be more interested and more engaged in smoking cessation activities compared to the general population. In addition, tobacco consumption will be measured via questionnaire, and consequently, patients may over- or underreport the amount of tobacco consumed. However, measuring tobacco consumption via self-report measures is generally accurate and is most appropriate because the study design does not allow for biochemical validation [35]. A possible source of bias is that patients will be recruited after medical practices have been randomly allocated to the intervention or control group. In this case, the allocation schedule is known, which may lead to biased participant recruitment within medical practices [36].

### Barriers to study completion

Possible barriers to the study’s implementation are seen primarily in the willingness of physicians to participate in research. However, this barrier will be addressed by actively following up on invitation letters with no response via telephone, engaging medical assistants in study processes and assuring adequate reimbursement in addition to training physicians individually. Despite these precautions drop-out is expected, specifically for medical practices that are assigned to the control group. However, the expected drop-out has been taken into account during sample size calculation. Another concern regarding the study’s implementation is seen in the low response rate of surveys. This concern will be addressed by conducting follow-up surveys via telephone because these have previously resulted in response rates of up to 85 % in studies to evaluate the program “Rauchfrei” by IFT [11].

## Trial status

Participant recruitment started in June 2014 and is expected to be completed in September 2015. This is the first version of the study protocol and no amendments have been made to the study design.

## Abbreviations

CG: Control group
COPD: Chronic obstructive pulmonary disease
CSCI: Collaborative smoking cessation intervention
DRKS: Deutsches Register Klinischer Studien (German Clinical Trials Register)
ENTER: Evaluation of a collaborative smoking cessation intervention
EQ-5D: European quality of life - Five dimensions health status questionnaire
FÄR: Fragebogen zur Änderungsbereitschaft bei Rauchern (Questionnaire to assess readiness to change among smokers)
FEV1: Forced expiratory volume in 1 second
FTND: Fagerström Test for Nicotine Dependence
GP: General practitioner
ICER: Incremental cost-effectiveness ratio
IFT: Institute for Therapy Research
IG: Intervention group
QALY: Quality-adjusted life year
SF-12: Short Form 12-item survey
VSRQ: Visual Simplified Respiratory Questionnaire
